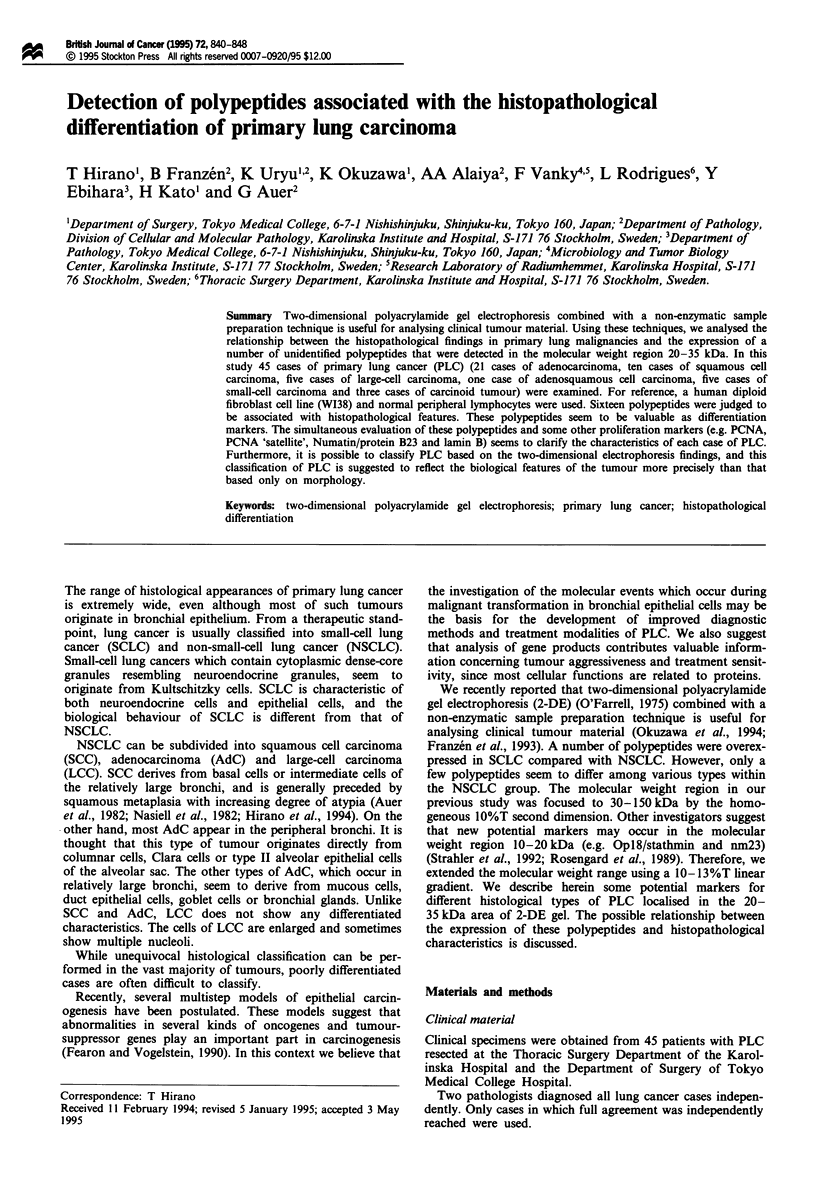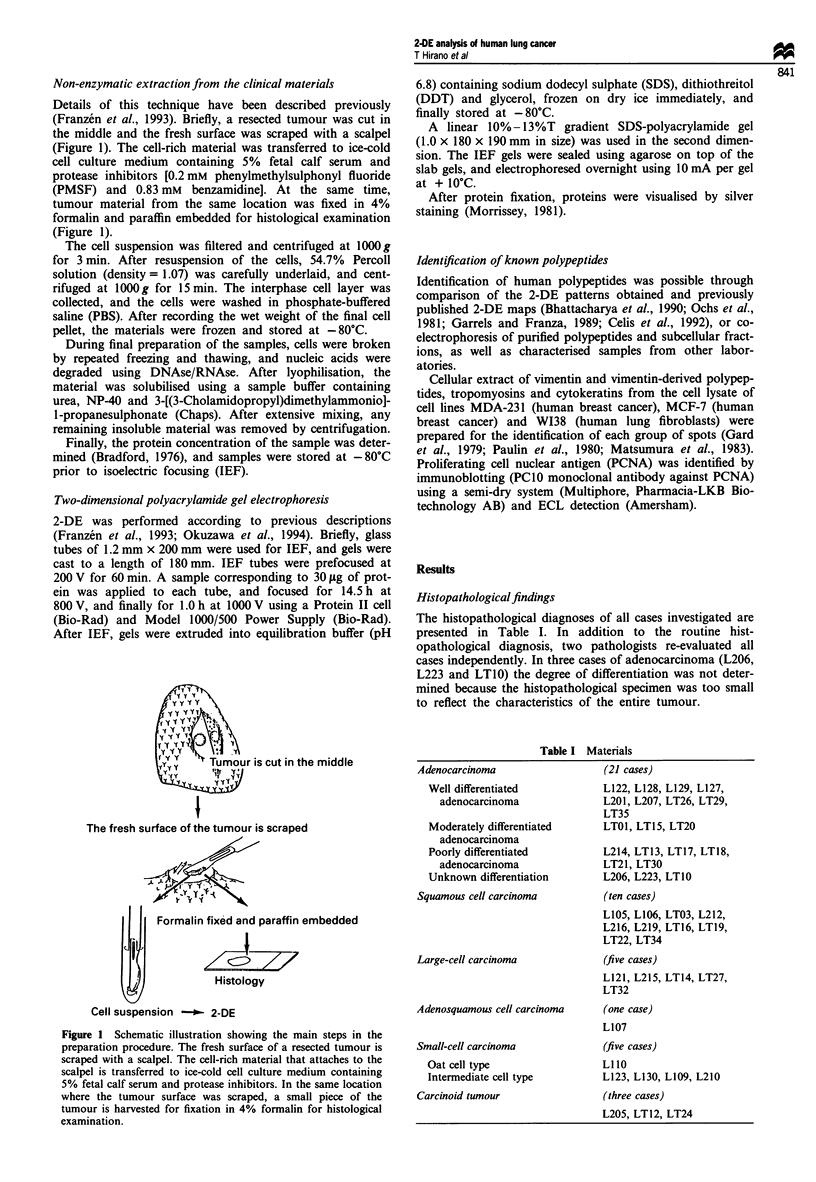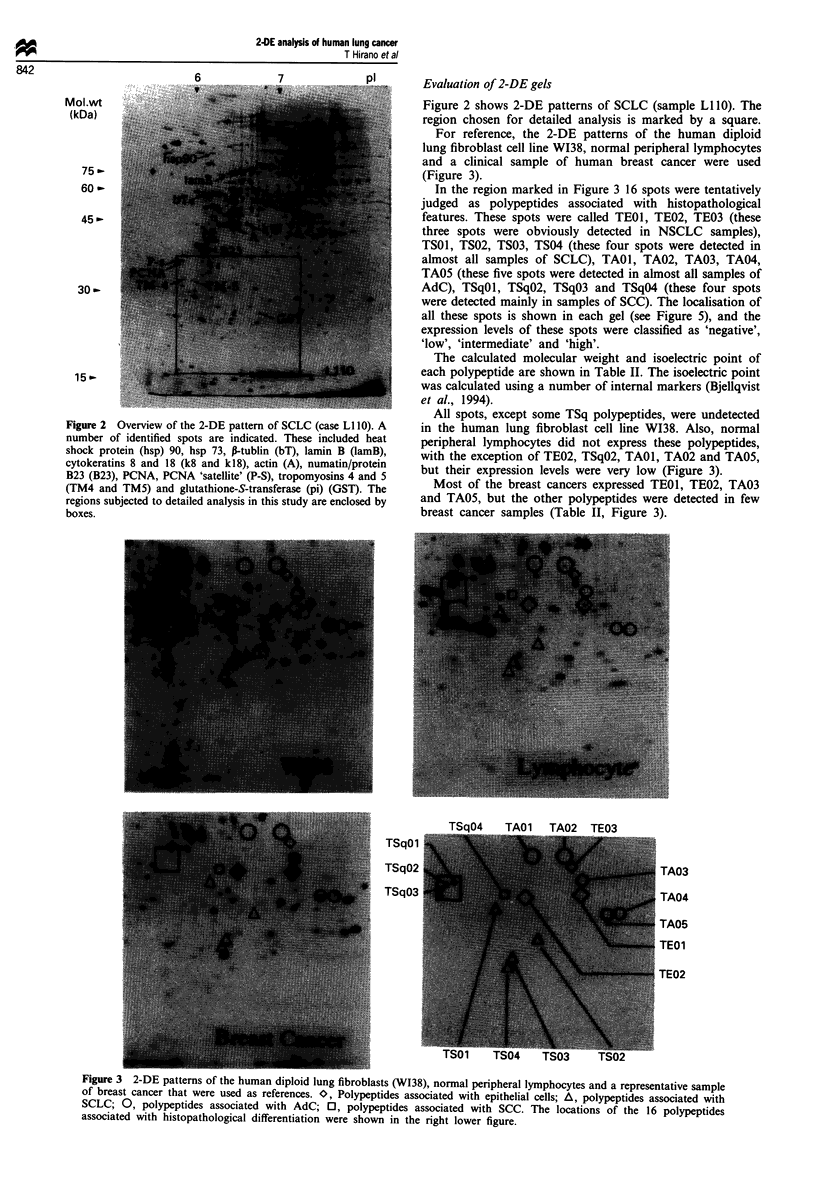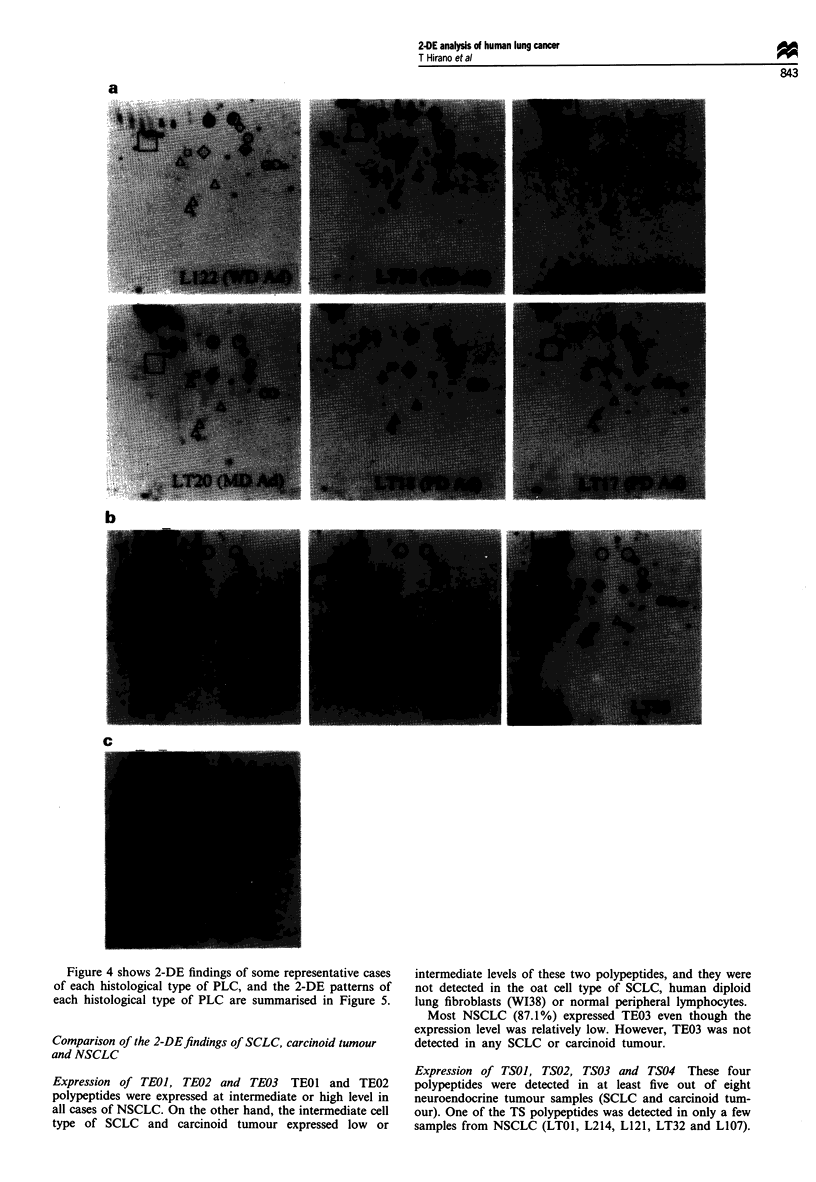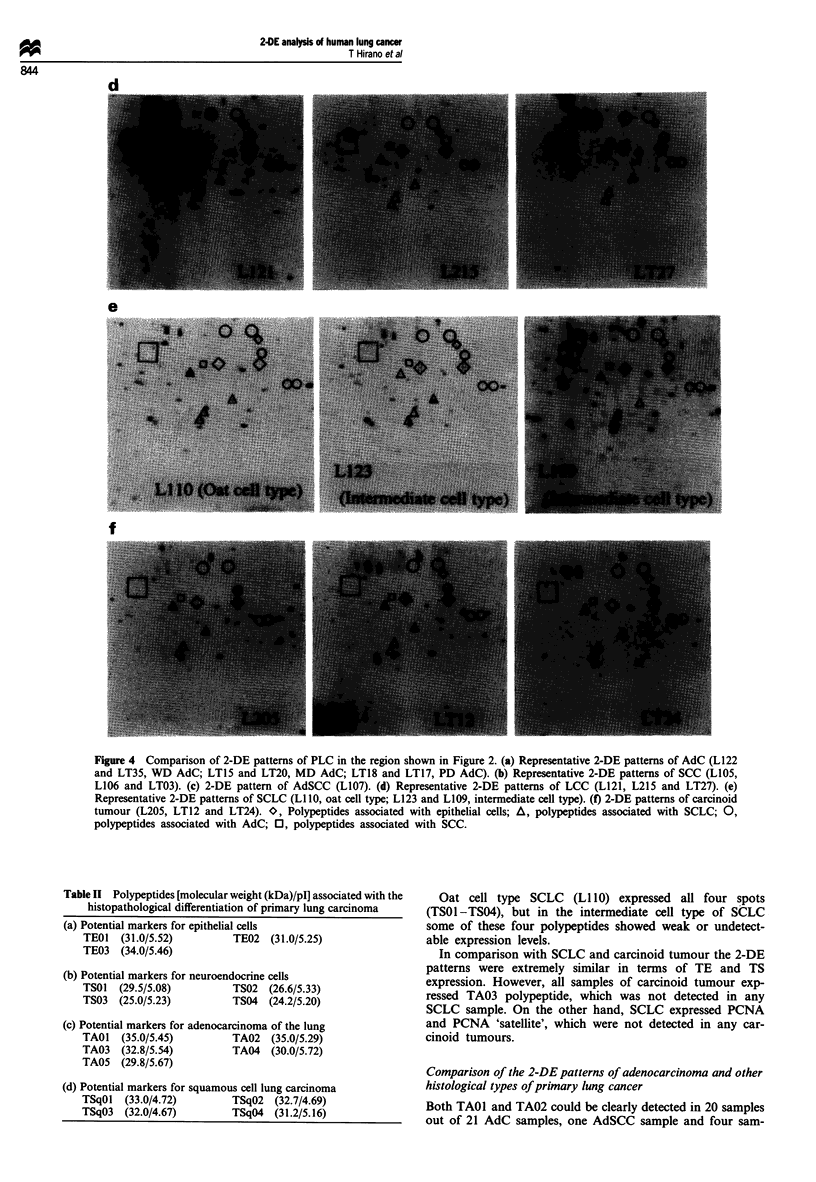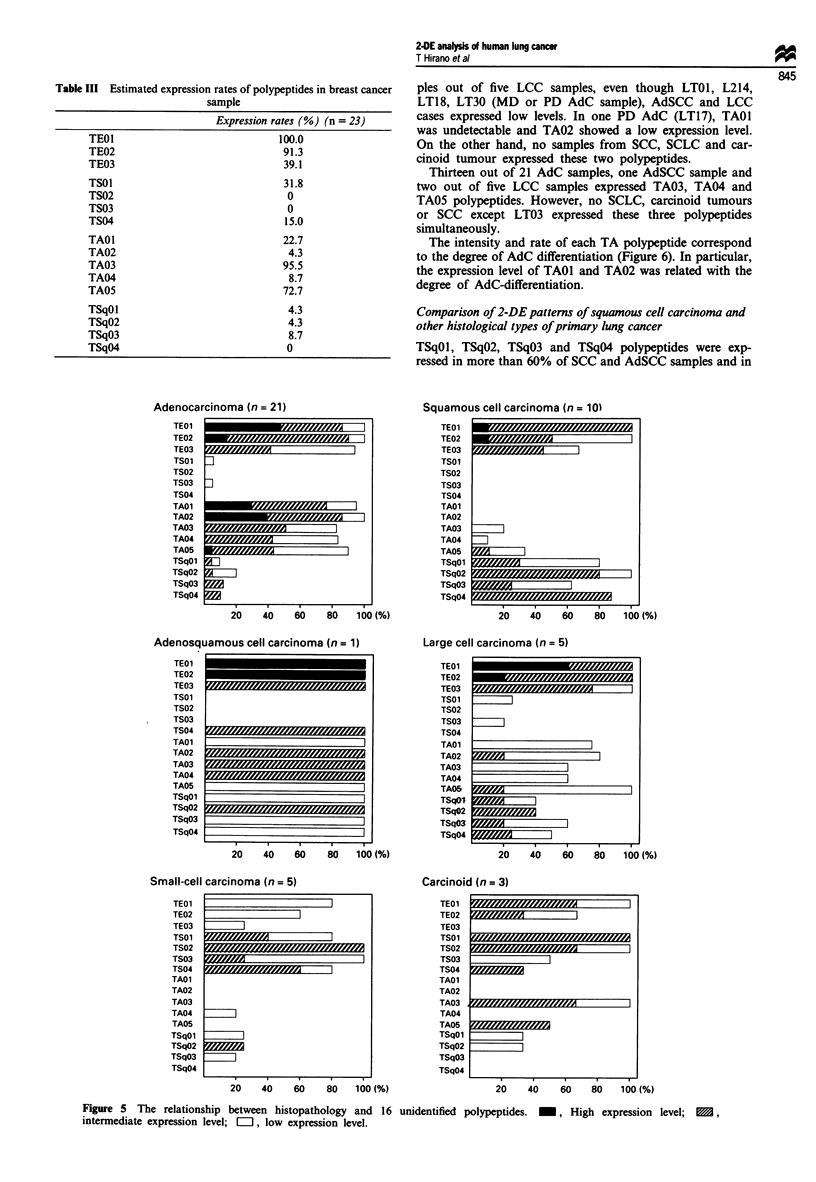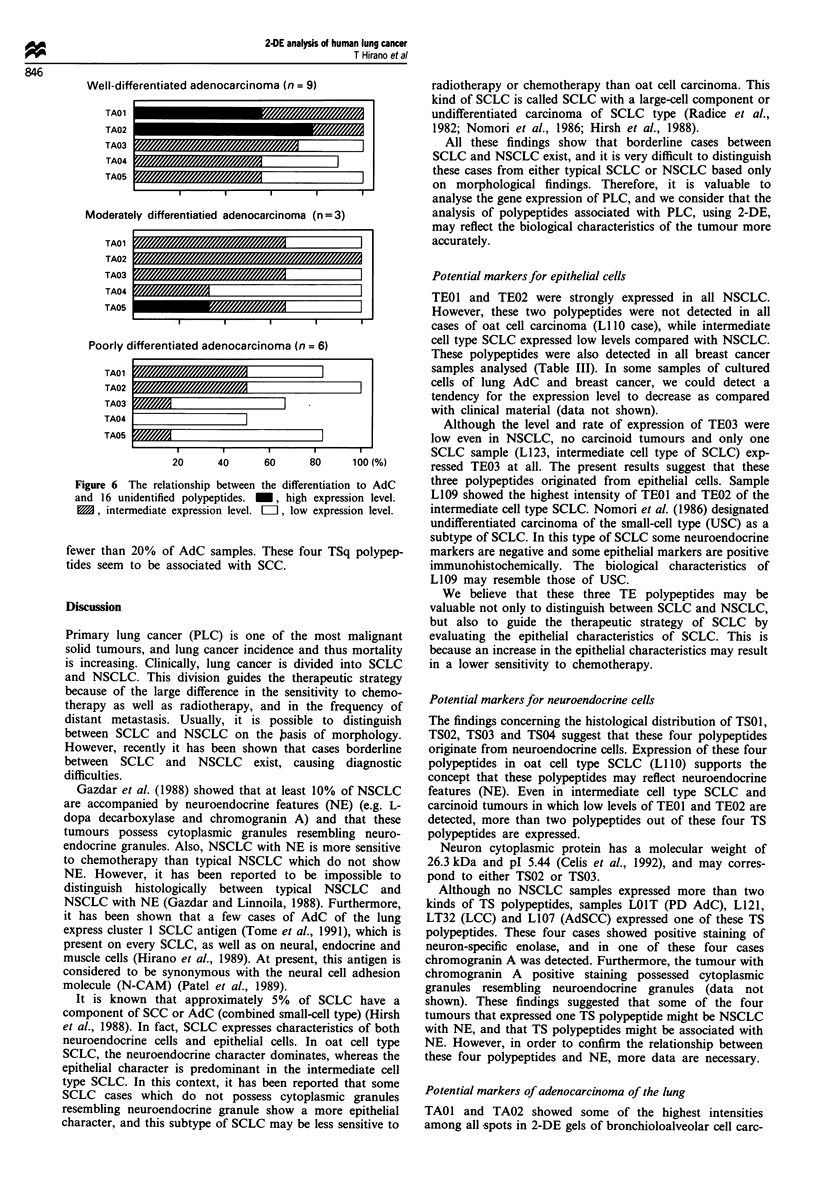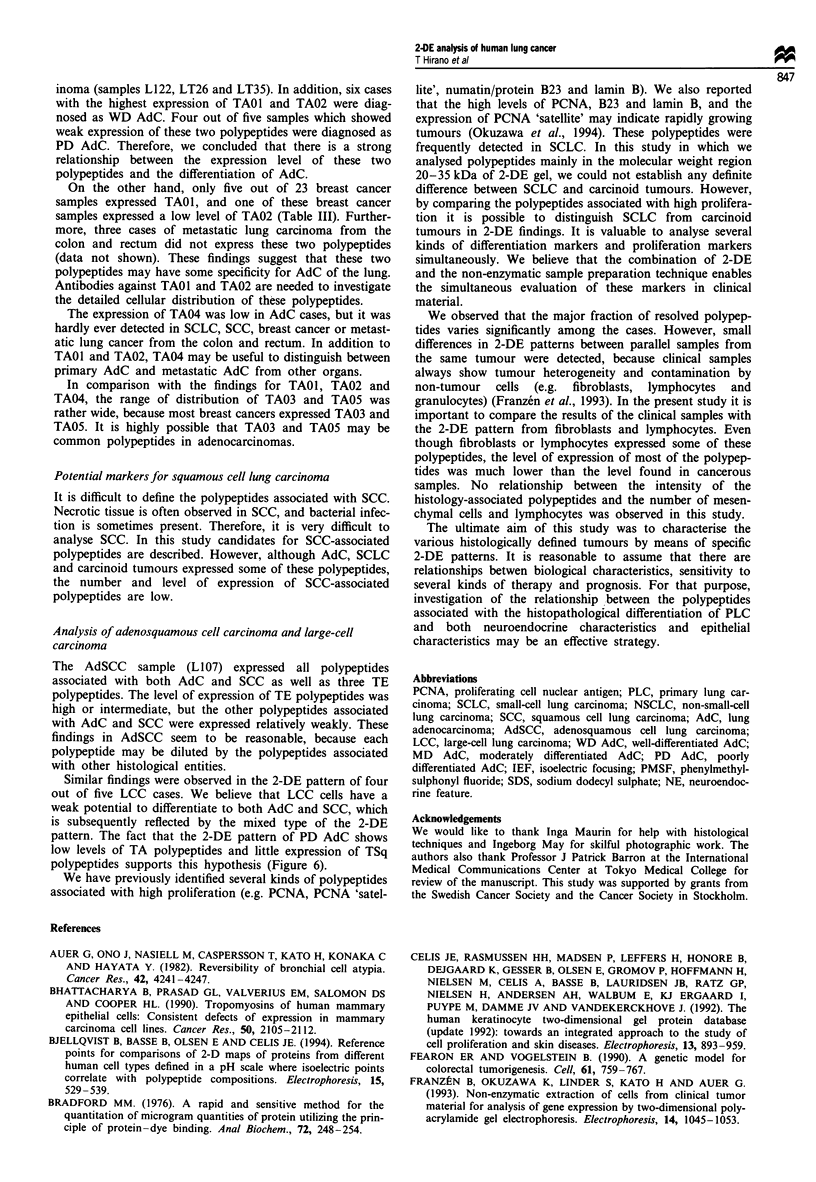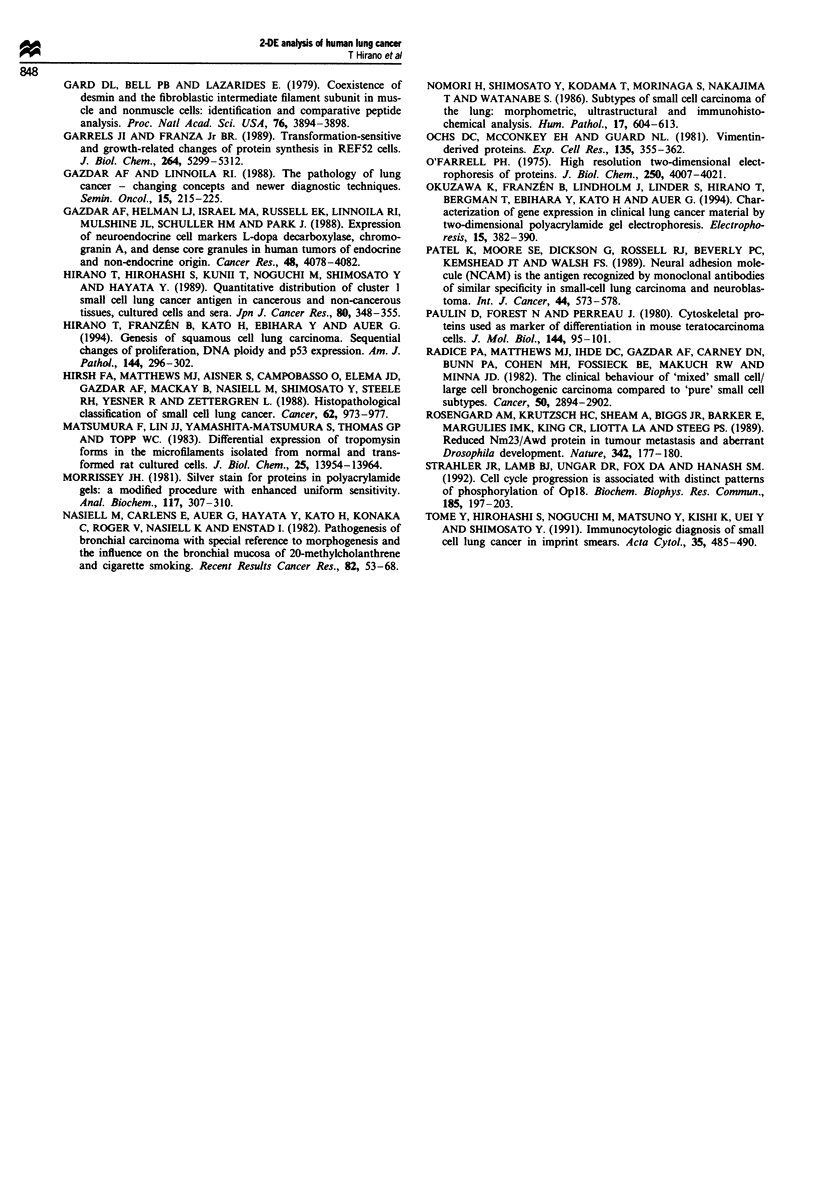# Detection of polypeptides associated with the histopathological differentiation of primary lung carcinoma.

**DOI:** 10.1038/bjc.1995.422

**Published:** 1995-10

**Authors:** T. Hirano, B. Franzén, K. Uryu, K. Okuzawa, A. A. Alaiya, F. Vanky, L. Rodrigues, Y. Ebihara, H. Kato, G. Auer

**Affiliations:** Department of Surgery, Tokyo Medical College, Japan.

## Abstract

**Images:**


British Journal of Cancer (1995) 72, 840-848
© 1995 Stockton Press All rights reserved 0007-0920/95 $12.00

### Detection of polypeptides associated with the histopathological differentiation of primary lung carcinoma

T Hirano[1], B Franzén[2], K Uryu[1,2], K Okuzawa[1], AA Alaiya[2], F Vanky[4,5], L Rodrigues[6], Y Ebihara[3], H Kato[1] and G Auer[2]

[1]*Department of Surgery, Tokyo Medical College, 6-7-1 Nishishinjuku, Shinjuku-ku, Tokyo 160, Japan;* [2]*Department of Pathology, Division of Cellular and Molecular Pathology, Karolinska Institute and Hospital, S-171 76 Stockholm, Sweden;* [3]*Department of Pathology, Tokyo Medical College, 6-7-1 Nishishinjuku, Shinjuku-ku, Tokyo 160, Japan;* [4]*Microbiology and Tumor Biology Center, Karolinska Institute, S-171 77 Stockholm, Sweden;* [5]*Research Laboratory of Radiumhemmet, Karolinska Hospital, S-171 76 Stockholm, Sweden;* [6]*Thoracic Surgery Department, Karolinska Institute and Hospital, S-171 76 Stockholm, Sweden.*

**Summary** Two-dimensional polyacrylamide gel electrophoresis combined with a non-enzymatic sample preparation technique is useful for analysing clinical tumour material. Using these techniques, we analysed the relationship between the histopathological findings in primary lung malignancies and the expression of a number of unidentified polypeptides that were detected in the molecular weight region 20–35 kDa. In this study 45 cases of primary lung cancer (PLC) (21 cases of adenocarcinoma, ten cases of squamous cell carcinoma, five cases of large-cell carcinoma, one case of adenosquamous cell carcinoma, five cases of small-cell carcinoma and three cases of carcinoid tumour) were examined. For reference, a human diploid fibroblast cell line (WI38) and normal peripheral lymphocytes were used. Sixteen polypeptides were judged to be associated with histopathological features. These polypeptides seem to be valuable as differentiation markers. The simultaneous evaluation of these polypeptides and some other proliferation markers (e.g. PCNA, PCNA 'satellite', Numatin/protein B23 and lamin B) seems to clarify the characteristics of each case of PLC. Furthermore, it is possible to classify PLC based on the two-dimensional electrophoresis findings, and this classification of PLC is suggested to reflect the biological features of the tumour more precisely than that based only on morphology.

**Keywords:** two-dimensional polyacrylamide gel electrophoresis; primary lung cancer; histopathological differentiation

The range of histological appearances of primary lung cancer is extremely wide, even although most of such tumours originate in bronchial epithelium. From a therapeutic standpoint, lung cancer is usually classified into small-cell lung cancer (SCLC) and non-small-cell lung cancer (NSCLC). Small-cell lung cancers which contain cytoplasmic dense-core granules resembling neuroendocrine granules, seem to originate from Kultschitzky cells. SCLC is characteristic of both neuroendocrine cells and epithelial cells, and the biological behaviour of SCLC is different from that of NSCLC.

NSCLC can be subdivided into squamous cell carcinoma (SCC), adenocarcinoma (AdC) and large-cell carcinoma (LCC). SCC derives from basal cells or intermediate cells of the relatively large bronchi, and is generally preceded by squamous metaplasia with increasing degree of atypia (Auer *et al.*, 1982; Nasiell *et al.*, 1982; Hirano *et al.*, 1994). On the other hand, most AdC appear in the peripheral bronchi. It is thought that this type of tumour originates directly from columnar cells, Clara cells or type II alveolar epithelial cells of the alveolar sac. The other types of AdC, which occur in relatively large bronchi, seem to derive from mucous cells, duct epithelial cells, goblet cells or bronchial glands. Unlike SCC and AdC, LCC does not show any differentiated characteristics. The cells of LCC are enlarged and sometimes show multiple nucleoli.

While unequivocal histological classification can be performed in the vast majority of tumours, poorly differentiated cases are often difficult to classify.

Recently, several multistep models of epithelial carcinogenesis have been postulated. These models suggest that abnormalities in several kinds of oncogenes and tumour-suppressor genes play an important part in carcinogenesis (Fearon and Vogelstein, 1990). In this context we believe that the investigation of the molecular events which occur during malignant transformation in bronchial epithelial cells may be the basis for the development of improved diagnostic methods and treatment modalities of PLC. We also suggest that analysis of gene products contributes valuable information concerning tumour aggressiveness and treatment sensitivity, since most cellular functions are related to proteins.

We recently reported that two-dimensional polyacrylamide gel electrophoresis (2-DE) (O'Farrell, 1975) combined with a non-enzymatic sample preparation technique is useful for analysing clinical tumour material (Okuzawa *et al.*, 1994; Franzén *et al.*, 1993). A number of polypeptides were overexpressed in SCLC compared with NSCLC. However, only a few polypeptides seem to differ among various types within the NSCLC group. The molecular weight region in our previous study was focused to 30–150 kDa by the homogeneous 10%T second dimension. Other investigators suggest that new potential markers may occur in the molecular weight region 10–20 kDa (e.g. Op18/stathmin and nm23) (Strahler *et al.*, 1992; Rosengard *et al.*, 1989). Therefore, we extended the molecular weight range using a 10–13%T linear gradient. We describe herein some potential markers for different histological types of PLC localised in the 20–35 kDa area of 2-DE gel. The possible relationship between the expression of these polypeptides and histopathological characteristics is discussed.

### Materials and methods

### *Clinical material*

Clinical specimens were obtained from 45 patients with PLC resected at the Thoracic Surgery Department of the Karolinska Hospital and the Department of Surgery of Tokyo Medical College Hospital.

Two pathologists diagnosed all lung cancer cases independently. Only cases in which full agreement was independently reached were used.

Correspondence: T Hirano
Received 11 February 1994; revised 5 January 1995; accepted 3 May 1995

*Non-enzymatic extraction from the clinical materials*

Details of this technique have been described previously (Franzén *et al.*, 1993). Briefly, a resected tumour was cut in the middle and the fresh surface was scraped with a scalpel (Figure 1). The cell-rich material was transferred to ice-cold cell culture medium containing 5% fetal calf serum and protease inhibitors [0.2 mM phenylmethylsulphonyl fluoride (PMSF) and 0.83 mM benzamidine]. At the same time, tumour material from the same location was fixed in 4% formalin and paraffin embedded for histological examination (Figure 1).

The cell suspension was filtered and centrifuged at 1000 *g* for 3 min. After resuspension of the cells, 54.7% Percoll solution (density = 1.07) was carefully underlaid, and centrifuged at 1000 *g* for 15 min. The interphase cell layer was collected, and the cells were washed in phosphate-buffered saline (PBS). After recording the wet weight of the final cell pellet, the materials were frozen and stored at − 80°C.

During final preparation of the samples, cells were broken by repeated freezing and thawing, and nucleic acids were degraded using DNAse/RNAse. After lyophilisation, the material was solubilised using a sample buffer containing urea, NP-40 and 3-[(3-Cholamidopropyl)dimethylammonio]-1-propanesulphonate (Chaps). After extensive mixing, any remaining insoluble material was removed by centrifugation.

Finally, the protein concentration of the sample was determined (Bradford, 1976), and samples were stored at − 80°C prior to isoelectric focusing (IEF).

*Two-dimensional polyacrylamide gel electrophoresis*

2-DE was performed according to previous descriptions (Franzén *et al.*, 1993; Okuzawa *et al.*, 1994). Briefly, glass tubes of 1.2 mm × 200 mm were used for IEF, and gels were cast to a length of 180 mm. IEF tubes were prefocused at 200 V for 60 min. A sample corresponding to 30 μg of protein was applied to each tube, and focused for 14.5 h at 800 V, and finally for 1.0 h at 1000 V using a Protein II cell (Bio-Rad) and Model 1000/500 Power Supply (Bio-Rad). After IEF, gels were extruded into equilibration buffer (pH 6.8) containing sodium dodecyl sulphate (SDS), dithiothreitol (DDT) and glycerol, frozen on dry ice immediately, and finally stored at − 80°C.

A linear 10%–13%T gradient SDS-polyacrylamide gel (1.0 × 180 × 190 mm in size) was used in the second dimension. The IEF gels were sealed using agarose on top of the slab gels, and electrophoresed overnight using 10 mA per gel at + 10°C.

After protein fixation, proteins were visualised by silver staining (Morrissey, 1981).

**Figure 1** Schematic illustration showing the main steps in the preparation procedure. The fresh surface of a resected tumour is scraped with a scalpel. The cell-rich material that attaches to the scalpel is transferred to ice-cold cell culture medium containing 5% fetal calf serum and protease inhibitors. In the same location where the tumour surface was scraped, a small piece of the tumour is harvested for fixation in 4% formalin for histological examination.

*Identification of known polypeptides*

Identification of human polypeptides was possible through comparison of the 2-DE patterns obtained and previously published 2-DE maps (Bhattacharya *et al.*, 1990; Ochs *et al.*, 1981; Garrels and Franza, 1989; Celis *et al.*, 1992), or co-electrophoresis of purified polypeptides and subcellular fractions, as well as characterised samples from other laboratories.

Cellular extract of vimentin and vimentin-derived polypeptides, tropomyosins and cytokeratins from the cell lysate of cell lines MDA-231 (human breast cancer), MCF-7 (human breast cancer) and WI38 (human lung fibroblasts) were prepared for the identification of each group of spots (Gard *et al.*, 1979; Paulin *et al.*, 1980; Matsumura *et al.*, 1983). Proliferating cell nuclear antigen (PCNA) was identified by immunoblotting (PC10 monoclonal antibody against PCNA) using a semi-dry system (Multiphore, Pharmacia-LKB Biotechnology AB) and ECL detection (Amersham).

### Results

*Histopathological findings*

The histopathological diagnoses of all cases investigated are presented in Table I. In addition to the routine histopathological diagnosis, two pathologists re-evaluated all cases independently. In three cases of adenocarcinoma (L206, L223 and LT10) the degree of differentiation was not determined because the histopathological specimen was too small to reflect the characteristics of the entire tumour.

**Table I** Materials

| | |
|---|---|
| *Adenocarcinoma* | *(21 cases)* |
| Well differentiated adenocarcinoma | L122, L128, L129, L127, L201, L207, LT26, LT29, LT35 |
| Moderately differentiated adenocarcinoma | LT01, LT15, LT20 |
| Poorly differentiated adenocarcinoma | L214, LT13, LT17, LT18, LT21, LT30 |
| Unknown differentiation | L206, L223, LT10 |
| *Squamous cell carcinoma* | *(ten cases)* |
| | L105, L106, LT03, L212, L216, L219, LT16, LT19, LT22, LT34 |
| *Large-cell carcinoma* | *(five cases)* |
| | L121, L215, LT14, LT27, LT32 |
| *Adenosquamous cell carcinoma* | *(one case)* |
| | L107 |
| *Small-cell carcinoma* | *(five cases)* |
| Oat cell type | L110 |
| Intermediate cell type | L123, L130, L109, L210 |
| *Carcinoid tumour* | *(three cases)* |
| | L205, LT12, LT24 |

**Figure 2** Overview of the 2-DE pattern of SCLC (case L110). A number of identified spots are indicated. These included heat shock protein (hsp) 90, hsp 73, β-tublin (bT), lamin B (lamB), cytokeratins 8 and 18 (k8 and k18), actin (A), numatin/protein B23 (B23), PCNA, PCNA 'satellite' (P-S), tropomyosins 4 and 5 (TM4 and TM5) and glutathione-*S*-transferase (pi) (GST). The regions subjected to detailed analysis in this study are enclosed by boxes.

*Evaluation of 2-DE gels*

Figure 2 shows 2-DE patterns of SCLC (sample L110). The region chosen for detailed analysis is marked by a square.

For reference, the 2-DE patterns of the human diploid lung fibroblast cell line WI38, normal peripheral lymphocytes and a clinical sample of human breast cancer were used (Figure 3).

In the region marked in Figure 3 16 spots were tentatively judged as polypeptides associated with histopathological features. These spots were called TE01, TE02, TE03 (these three spots were obviously detected in NSCLC samples), TS01, TS02, TS03, TS04 (these four spots were detected in almost all samples of SCLC), TA01, TA02, TA03, TA04, TA05 (these five spots were detected in almost all samples of AdC), TSq01, TSq02, TSq03 and TSq04 (these four spots were detected mainly in samples of SCC). The localisation of all these spots is shown in each gel (see Figure 5), and the expression levels of these spots were classified as 'negative', 'low', 'intermediate' and 'high'.

The calculated molecular weight and isoelectric point of each polypeptide are shown in Table II. The isoelectric point was calculated using a number of internal markers (Bjellqvist *et al.*, 1994).

All spots, except some TSq polypeptides, were undetected in the human lung fibroblast cell line WI38. Also, normal peripheral lymphocytes did not express these polypeptides, with the exception of TE02, TSq02, TA01, TA02 and TA05, but their expression levels were very low (Figure 3).

Most of the breast cancers expressed TE01, TE02, TA03 and TA05, but the other polypeptides were detected in few breast cancer samples (Table II, Figure 3).

**Figure 3** 2-DE patterns of the human diploid lung fibroblasts (WI38), normal peripheral lymphocytes and a representative sample of breast cancer that were used as references. ◇, Polypeptides associated with epithelial cells; △, polypeptides associated with SCLC; ○, polypeptides associated with AdC; □, polypeptides associated with SCC. The locations of the 16 polypeptides associated with histopathological differentiation were shown in the right lower figure.

Figure 4 shows 2-DE findings of some representative cases of each histological type of PLC, and the 2-DE patterns of each histological type of PLC are summarised in Figure 5.

*Comparison of the 2-DE findings of SCLC, carcinoid tumour and NSCLC*

*Expression of TE01, TE02 and TE03* TE01 and TE02 polypeptides were expressed at intermediate or high level in all cases of NSCLC. On the other hand, the intermediate cell type of SCLC and carcinoid tumour expressed low or intermediate levels of these two polypeptides, and they were not detected in the oat cell type of SCLC, human diploid lung fibroblasts (WI38) or normal peripheral lymphocytes.

Most NSCLC (87.1%) expressed TE03 even though the expression level was relatively low. However, TE03 was not detected in any SCLC or carcinoid tumour.

*Expression of TS01, TS02, TS03 and TS04* These four polypeptides were detected in at least five out of eight neuroendocrine tumour samples (SCLC and carcinoid tumour). One of the TS polypeptides was detected in only a few samples from NSCLC (LT01, L214, L121, LT32 and L107).

**Figure 4** Comparison of 2-DE patterns of PLC in the region shown in Figure 2. (**a**) Representative 2-DE patterns of AdC (L122 and LT35, WD AdC; LT15 and LT20, MD AdC; LT18 and LT17, PD AdC). (**b**) Representative 2-DE patterns of SCC (L105, L106 and LT03). (**c**) 2-DE pattern of AdSCC (L107). (**d**) Representative 2-DE patterns of LCC (L121, L215 and LT27). (**e**) Representative 2-DE patterns of SCLC (L110, oat cell type; L123 and L109, intermediate cell type). (**f**) 2-DE patterns of carcinoid tumour (L205, LT12 and LT24). ◇, Polypeptides associated with epithelial cells; △, polypeptides associated with SCLC; ○, polypeptides associated with AdC; □, polypeptides associated with SCC.

**Table II** Polypeptides [molecular weight (kDa)/pI] associated with the histopathological differentiation of primary lung carcinoma

| | | | |
|---|---|---|---|
| (a) Potential markers for epithelial cells | | | |
| TE01 | (31.0/5.52) | TE02 | (31.0/5.25) |
| TE03 | (34.0/5.46) | | |
| (b) Potential markers for neuroendocrine cells | | | |
| TS01 | (29.5/5.08) | TS02 | (26.6/5.33) |
| TS03 | (25.0/5.23) | TS04 | (24.2/5.20) |
| (c) Potential markers for adenocarcinoma of the lung | | | |
| TA01 | (35.0/5.45) | TA02 | (35.0/5.29) |
| TA03 | (32.8/5.54) | TA04 | (30.0/5.72) |
| TA05 | (29.8/5.67) | | |
| (d) Potential markers for squamous cell lung carcinoma | | | |
| TSq01 | (33.0/4.72) | TSq02 | (32.7/4.69) |
| TSq03 | (32.0/4.67) | TSq04 | (31.2/5.16) |

Oat cell type SCLC (L110) expressed all four spots (TS01–TS04), but in the intermediate cell type of SCLC some of these four polypeptides showed weak or undetectable expression levels.

In comparison with SCLC and carcinoid tumour the 2-DE patterns were extremely similar in terms of TE and TS expression. However, all samples of carcinoid tumour expressed TA03 polypeptide, which was not detected in any SCLC sample. On the other hand, SCLC expressed PCNA and PCNA 'satellite', which were not detected in any carcinoid tumours.

*Comparison of the 2-DE patterns of adenocarcinoma and other histological types of primary lung cancer*

Both TA01 and TA02 could be clearly detected in 20 samples out of 21 AdC samples, one AdSCC sample and four sam-

**Table III** Estimated expression rates of polypeptides in breast cancer sample

| | *Expression rates (%) (n = 23)* |
|---|---|
| TE01 | 100.0 |
| TE02 | 91.3 |
| TE03 | 39.1 |
| TS01 | 31.8 |
| TS02 | 0 |
| TS03 | 0 |
| TS04 | 15.0 |
| TA01 | 22.7 |
| TA02 | 4.3 |
| TA03 | 95.5 |
| TA04 | 8.7 |
| TA05 | 72.7 |
| TSq01 | 4.3 |
| TSq02 | 4.3 |
| TSq03 | 8.7 |
| TSq04 | 0 |

ples out of five LCC samples, even though LT01, L214, LT18, LT30 (MD or PD AdC sample), AdSCC and LCC cases expressed low levels. In one PD AdC (LT17), TA01 was undetectable and TA02 showed a low expression level. On the other hand, no samples from SCC, SCLC and carcinoid tumour expressed these two polypeptides.

Thirteen out of 21 AdC samples, one AdSCC sample and two out of five LCC samples expressed TA03, TA04 and TA05 polypeptides. However, no SCLC, carcinoid tumours or SCC except LT03 expressed these three polypeptides simultaneously.

The intensity and rate of each TA polypeptide correspond to the degree of AdC differentiation (Figure 6). In particular, the expression level of TA01 and TA02 was related with the degree of AdC-differentiation.

### *Comparison of 2-DE patterns of squamous cell carcinoma and other histological types of primary lung cancer*

TSq01, TSq02, TSq03 and TSq04 polypeptides were expressed in more than 60% of SCC and AdSCC samples and in

**Figure 5** The relationship between histopathology and 16 unidentified polypeptides. ■, High expression level; ▨, intermediate expression level; □, low expression level.

**Figure 6** The relationship between the differentiation to AdC and 16 unidentified polypeptides. ■, high expression level. ▨, intermediate expression level. □, low expression level.

fewer than 20% of AdC samples. These four TSq polypeptides seem to be associated with SCC.

### Discussion

Primary lung cancer (PLC) is one of the most malignant solid tumours, and lung cancer incidence and thus mortality is increasing. Clinically, lung cancer is divided into SCLC and NSCLC. This division guides the therapeutic strategy because of the large difference in the sensitivity to chemotherapy as well as radiotherapy, and in the frequency of distant metastasis. Usually, it is possible to distinguish between SCLC and NSCLC on the basis of morphology. However, recently it has been shown that cases borderline between SCLC and NSCLC exist, causing diagnostic difficulties.

Gazdar *et al.* (1988) showed that at least 10% of NSCLC are accompanied by neuroendocrine features (NE) (e.g. L-dopa decarboxylase and chromogranin A) and that these tumours possess cytoplasmic granules resembling neuroendocrine granules. Also, NSCLC with NE is more sensitive to chemotherapy than typical NSCLC which do not show NE. However, it has been reported to be impossible to distinguish histologically between typical NSCLC and NSCLC with NE (Gazdar and Linnoila, 1988). Furthermore, it has been shown that a few cases of AdC of the lung express cluster 1 SCLC antigen (Tome *et al.*, 1991), which is present on every SCLC, as well as on neural, endocrine and muscle cells (Hirano *et al.*, 1989). At present, this antigen is considered to be synonymous with the neural cell adhesion molecule (N-CAM) (Patel *et al.*, 1989).

It is known that approximately 5% of SCLC have a component of SCC or AdC (combined small-cell type) (Hirsh *et al.*, 1988). In fact, SCLC expresses characteristics of both neuroendocrine cells and epithelial cells. In oat cell type SCLC, the neuroendocrine character dominates, whereas the epithelial character is predominant in the intermediate cell type SCLC. In this context, it has been reported that some SCLC cases which do not possess cytoplasmic granules resembling neuroendocrine granule show a more epithelial character, and this subtype of SCLC may be less sensitive to radiotherapy or chemotherapy than oat cell carcinoma. This kind of SCLC is called SCLC with a large-cell component or undifferentiated carcinoma of SCLC type (Radice *et al.*, 1982; Nomori *et al.*, 1986; Hirsh *et al.*, 1988).

All these findings show that borderline cases between SCLC and NSCLC exist, and it is very difficult to distinguish these cases from either typical SCLC or NSCLC based only on morphological findings. Therefore, it is valuable to analyse the gene expression of PLC, and we consider that the analysis of polypeptides associated with PLC, using 2-DE, may reflect the biological characteristics of the tumour more accurately.

### *Potential markers for epithelial cells*

TE01 and TE02 were strongly expressed in all NSCLC. However, these two polypeptides were not detected in all cases of oat cell carcinoma (L110 case), while intermediate cell type SCLC expressed low levels compared with NSCLC. These polypeptides were also detected in all breast cancer samples analysed (Table III). In some samples of cultured cells of lung AdC and breast cancer, we could detect a tendency for the expression level to decrease as compared with clinical material (data not shown).

Although the level and rate of expression of TE03 were low even in NSCLC, no carcinoid tumours and only one SCLC sample (L123, intermediate cell type of SCLC) expressed TE03 at all. The present results suggest that these three polypeptides originated from epithelial cells. Sample L109 showed the highest intensity of TE01 and TE02 of the intermediate cell type SCLC. Nomori *et al.* (1986) designated undifferentiated carcinoma of the small-cell type (USC) as a subtype of SCLC. In this type of SCLC some neuroendocrine markers are negative and some epithelial markers are positive immunohistochemically. The biological characteristics of L109 may resemble those of USC.

We believe that these three TE polypeptides may be valuable not only to distinguish between SCLC and NSCLC, but also to guide the therapeutic strategy of SCLC by evaluating the epithelial characteristics of SCLC. This is because an increase in the epithelial characteristics may result in a lower sensitivity to chemotherapy.

### *Potential markers for neuroendocrine cells*

The findings concerning the histological distribution of TS01, TS02, TS03 and TS04 suggest that these four polypeptides originate from neuroendocrine cells. Expression of these four polypeptides in oat cell type SCLC (L110) supports the concept that these polypeptides may reflect neuroendocrine features (NE). Even in intermediate cell type SCLC and carcinoid tumours in which low levels of TE01 and TE02 are detected, more than two polypeptides out of these four TS polypeptides are expressed.

Neuron cytoplasmic protein has a molecular weight of 26.3 kDa and pI 5.44 (Celis *et al.*, 1992), and may correspond to either TS02 or TS03.

Although no NSCLC samples expressed more than two kinds of TS polypeptides, samples L01T (PD AdC), L121, LT32 (LCC) and L107 (AdSCC) expressed one of these TS polypeptides. These four cases showed positive staining of neuron-specific enolase, and in one of these four cases chromogranin A was detected. Furthermore, the tumour with chromogranin A positive staining possessed cytoplasmic granules resembling neuroendocrine granules (data not shown). These findings suggested that some of the four tumours that expressed one TS polypeptide might be NSCLC with NE, and that TS polypeptides might be associated with NE. However, in order to confirm the relationship between these four polypeptides and NE, more data are necessary.

### *Potential markers of adenocarcinoma of the lung*

TA01 and TA02 showed some of the highest intensities among all spots in 2-DE gels of bronchioloalveolar cell carcinoma (samples L122, LT26 and LT35). In addition, six cases with the highest expression of TA01 and TA02 were diagnosed as WD AdC. Four out of five samples which showed weak expression of these two polypeptides were diagnosed as PD AdC. Therefore, we concluded that there is a strong relationship between the expression level of these two polypeptides and the differentiation of AdC.

On the other hand, only five out of 23 breast cancer samples expressed TA01, and one of these breast cancer samples expressed a low level of TA02 (Table III). Furthermore, three cases of metastatic lung carcinoma from the colon and rectum did not express these two polypeptides (data not shown). These findings suggest that these two polypeptides may have some specificity for AdC of the lung. Antibodies against TA01 and TA02 are needed to investigate the detailed cellular distribution of these polypeptides.

The expression of TA04 was low in AdC cases, but it was hardly ever detected in SCLC, SCC, breast cancer or metastatic lung cancer from the colon and rectum. In addition to TA01 and TA02, TA04 may be useful to distinguish between primary AdC and metastatic AdC from other organs.

In comparison with the findings for TA01, TA02 and TA04, the range of distribution of TA03 and TA05 was rather wide, because most breast cancers expressed TA03 and TA05. It is highly possible that TA03 and TA05 may be common polypeptides in adenocarcinomas.

*Potential markers for squamous cell lung carcinoma*

It is difficult to define the polypeptides associated with SCC. Necrotic tissue is often observed in SCC, and bacterial infection is sometimes present. Therefore, it is very difficult to analyse SCC. In this study candidates for SCC-associated polypeptides are described. However, although AdC, SCLC and carcinoid tumours expressed some of these polypeptides, the number and level of expression of SCC-associated polypeptides are low.

*Analysis of adenosquamous cell carcinoma and large-cell carcinoma*

The AdSCC sample (L107) expressed all polypeptides associated with both AdC and SCC as well as three TE polypeptides. The level of expression of TE polypeptides was high or intermediate, but the other polypeptides associated with AdC and SCC were expressed relatively weakly. These findings in AdSCC seem to be reasonable, because each polypeptide may be diluted by the polypeptides associated with other histological entities.

Similar findings were observed in the 2-DE pattern of four out of five LCC cases. We believe that LCC cells have a weak potential to differentiate to both AdC and SCC, which is subsequently reflected by the mixed type of the 2-DE pattern. The fact that the 2-DE pattern of PD AdC shows low levels of TA polypeptides and little expression of TSq polypeptides supports this hypothesis (Figure 6).

We have previously identified several kinds of polypeptides associated with high proliferation (e.g. PCNA, PCNA 'satellite', numatin/protein B23 and lamin B). We also reported that the high levels of PCNA, B23 and lamin B, and the expression of PCNA 'satellite' may indicate rapidly growing tumours (Okuzawa *et al.*, 1994). These polypeptides were frequently detected in SCLC. In this study in which we analysed polypeptides mainly in the molecular weight region 20–35 kDa of 2-DE gel, we could not establish any definite difference between SCLC and carcinoid tumours. However, by comparing the polypeptides associated with high proliferation it is possible to distinguish SCLC from carcinoid tumours in 2-DE findings. It is valuable to analyse several kinds of differentiation markers and proliferation markers simultaneously. We believe that the combination of 2-DE and the non-enzymatic sample preparation technique enables the simultaneous evaluation of these markers in clinical material.

We observed that the major fraction of resolved polypeptides varies significantly among the cases. However, small differences in 2-DE patterns between parallel samples from the same tumour were detected, because clinical samples always show tumour heterogeneity and contamination by non-tumour cells (e.g. fibroblasts, lymphocytes and granulocytes) (Franzén *et al.*, 1993). In the present study it is important to compare the results of the clinical samples with the 2-DE pattern from fibroblasts and lymphocytes. Even though fibroblasts or lymphocytes expressed some of these polypeptides, the level of expression of most of the polypeptides was much lower than the level found in cancerous samples. No relationship between the intensity of the histology-associated polypeptides and the number of mesenchymal cells and lymphocytes was observed in this study.

The ultimate aim of this study was to characterise the various histologically defined tumours by means of specific 2-DE patterns. It is reasonable to assume that there are relationships betwen biological characteristics, sensitivity to several kinds of therapy and prognosis. For that purpose, investigation of the relationship between the polypeptides associated with the histopathological differentiation of PLC and both neuroendocrine characteristics and epithelial characteristics may be an effective strategy.

**Abbreviations**

PCNA, proliferating cell nuclear antigen; PLC, primary lung carcinoma; SCLC, small-cell lung carcinoma; NSCLC, non-small-cell lung carcinoma; SCC, squamous cell lung carcinoma; AdC, lung adenocarcinoma; AdSCC, adenosquamous cell lung carcinoma; LCC, large-cell lung carcinoma; WD AdC, well-differentiated AdC; MD AdC, moderately differentiated AdC; PD AdC, poorly differentiated AdC; IEF, isoelectric focusing; PMSF, phenylmethylsulphonyl fluoride; SDS, sodium dodecyl sulphate; NE, neuroendocrine feature.

**Acknowledgements**

We would like to thank Inga Maurin for help with histological techniques and Ingeborg May for skilful photographic work. The authors also thank Professor J Patrick Barron at the International Medical Communications Center at Tokyo Medical College for review of the manuscript. This study was supported by grants from the Swedish Cancer Society and the Cancer Society in Stockholm.

**References**

AUER G, ONO J, NASIELL M, CASPERSSON T, KATO H, KONAKA C AND HAYATA Y. (1982). Reversibility of bronchial cell atypia. *Cancer Res.*, **42**, 4241–4247.

BHATTACHARYA B, PRASAD GL, VALVERIUS EM, SALOMON DS AND COOPER HL. (1990). Tropomyosins of human mammary epithelial cells: Consistent defects of expression in mammary carcinoma cell lines. *Cancer Res.*, **50**, 2105–2112.

BJELLQVIST B, BASSE B, OLSEN E AND CELIS JE. (1994). Reference points for comparisons of 2-D maps of proteins from different human cell types defined in a pH scale where isoelectric points correlate with polypeptide compositions. *Electrophoresis*, **15**, 529–539.

BRADFORD MM. (1976). A rapid and sensitive method for the quantitation of microgram quantities of protein utilizing the principle of protein–dye binding. *Anal Biochem.*, **72**, 248–254.

CELIS JE, RASMUSSEN HH, MADSEN P, LEFFERS H, HONORE B, DEJGAARD K, GESSER B, OLSEN E, GROMOV P, HOFFMANN H, NIELSEN M, CELIS A, BASSE B, LAURIDSEN JB, RATZ GP, NIELSEN H, ANDERSEN AH, WALBUM E, KJ ERGAARD I, PUYPE M, DAMME JV AND VANDEKERCKHOVE J. (1992). The human keratinocyte two-dimensional gel protein database (update 1992): towards an integrated approach to the study of cell proliferation and skin diseases. *Electrophoresis*, **13**, 893–959.

FEARON ER AND VOGELSTEIN B. (1990). A genetic model for colorectal tumorigenesis. *Cell*, **61**, 759–767.

FRANZÉN B, OKUZAWA K, LINDER S, KATO H AND AUER G. (1993). Non-enzymatic extraction of cells from clinical tumor material for analysis of gene expression by two-dimensional polyacrylamide gel electrophoresis. *Electrophoresis*, **14**, 1045–1053.

GARD DL, BELL PB AND LAZARIDES E. (1979). Coexistence of desmin and the fibroblastic intermediate filament subunit in muscle and nonmuscle cells: identification and comparative peptide analysis. *Proc. Natl Acad. Sci. USA*, **76**, 3894–3898.

GARRELS JI AND FRANZA Jr BR. (1989). Transformation-sensitive and growth-related changes of protein synthesis in REF52 cells. *J. Biol. Chem.*, **264**, 5299–5312.

GAZDAR AF AND LINNOILA RI. (1988). The pathology of lung cancer – changing concepts and newer diagnostic techniques. *Semin. Oncol.*, **15**, 215–225.

GAZDAR AF, HELMAN LJ, ISRAEL MA, RUSSELL EK, LINNOILA RI, MULSHINE JL, SCHULLER HM AND PARK J. (1988). Expression of neuroendocrine cell markers L-dopa decarboxylase, chromogranin A, and dense core granules in human tumors of endocrine and non-endocrine origin. *Cancer Res.*, **48**, 4078–4082.

HIRANO T, HIROHASHI S, KUNII T, NOGUCHI M, SHIMOSATO Y AND HAYATA Y. (1989). Quantitative distribution of cluster 1 small cell lung cancer antigen in cancerous and non-cancerous tissues, cultured cells and sera. *Jpn J. Cancer Res.*, **80**, 348–355.

HIRANO T, FRANZÉN B, KATO H, EBIHARA Y AND AUER G. (1994). Genesis of squamous cell lung carcinoma. Sequential changes of proliferation, DNA ploidy and p53 expression. *Am. J. Pathol.*, **144**, 296–302.

HIRSH FA, MATTHEWS MJ, AISNER S, CAMPOBASSO O, ELEMA JD, GAZDAR AF, MACKAY B, NASIELL M, SHIMOSATO Y, STEELE RH, YESNER R AND ZETTERGREN L. (1988). Histopathological classification of small cell lung cancer. *Cancer*, **62**, 973–977.

MATSUMURA F, LIN JJ, YAMASHITA-MATSUMURA S, THOMAS GP AND TOPP WC. (1983). Differential expression of tropomysin forms in the microfilaments isolated from normal and transformed rat cultured cells. *J. Biol. Chem.*, **25**, 13954–13964.

MORRISSEY JH. (1981). Silver stain for proteins in polyacrylamide gels: a modified procedure with enhanced uniform sensitivity. *Anal. Biochem.*, **117**, 307–310.

NASIELL M, CARLENS E, AUER G, HAYATA Y, KATO H, KONAKA C, ROGER V, NASIELL K AND ENSTAD I. (1982). Pathogenesis of bronchial carcinoma with special reference to morphogenesis and the influence on the bronchial mucosa of 20-methylcholanthrene and cigarette smoking. *Recent Results Cancer Res.*, **82**, 53–68.

NOMORI H, SHIMOSATO Y, KODAMA T, MORINAGA S, NAKAJIMA T AND WATANABE S. (1986). Subtypes of small cell carcinoma of the lung: morphometric, ultrastructural and immunohistochemical analysis. *Hum. Pathol.*, **17**, 604–613.

OCHS DC, MCCONKEY EH AND GUARD NL. (1981). Vimentin-derived proteins. *Exp. Cell Res.*, **135**, 355–362.

O'FARRELL PH. (1975). High resolution two-dimensional electrophoresis of proteins. *J. Biol. Chem.*, **250**, 4007–4021.

OKUZAWA K, FRANZÉN B, LINDHOLM J, LINDER S, HIRANO T, BERGMAN T, EBIHARA Y, KATO H AND AUER G. (1994). Characterization of gene expression in clinical lung cancer material by two-dimensional polyacrylamide gel electrophoresis. *Electrophoresis*, **15**, 382–390.

PATEL K, MOORE SE, DICKSON G, ROSSELL RJ, BEVERLY PC, KEMSHEAD JT AND WALSH FS. (1989). Neural adhesion molecule (NCAM) is the antigen recognized by monoclonal antibodies of similar specificity in small-cell lung carcinoma and neuroblastoma. *Int. J. Cancer*, **44**, 573–578.

PAULIN D, FOREST N AND PERREAU J. (1980). Cytoskeletal proteins used as marker of differentiation in mouse teratocarcinoma cells. *J. Mol. Biol.*, **144**, 95–101.

RADICE PA, MATTHEWS MJ, IHDE DC, GAZDAR AF, CARNEY DN, BUNN PA, COHEN MH, FOSSIECK BE, MAKUCH RW AND MINNA JD. (1982). The clinical behaviour of 'mixed' small cell/large cell bronchogenic carcinoma compared to 'pure' small cell subtypes. *Cancer*, **50**, 2894–2902.

ROSENGARD AM, KRUTZSCH HC, SHEAM A, BIGGS JR, BARKER E, MARGULIES IMK, KING CR, LIOTTA LA AND STEEG PS. (1989). Reduced Nm23/Awd protein in tumour metastasis and aberrant *Drosophila* development. *Nature*, **342**, 177–180.

STRAHLER JR, LAMB BJ, UNGAR DR, FOX DA AND HANASH SM. (1992). Cell cycle progression is associated with distinct patterns of phosphorylation of Op18. *Biochem. Biophys. Res. Commun.*, **185**, 197–203.

TOME Y, HIROHASHI S, NOGUCHI M, MATSUNO Y, KISHI K, UEI Y AND SHIMOSATO Y. (1991). Immunocytologic diagnosis of small cell lung cancer in imprint smears. *Acta Cytol.*, **35**, 485–490.